# Awareness of sustainability among gynecologists in Germany: results of a semirepresentative nationwide survey

**DOI:** 10.1007/s00404-026-08346-x

**Published:** 2026-02-20

**Authors:** Lina Judit Schiestl, Stefan Lukac, Carolin Hagedorn, Florian Ebner, Kerstin Bäumer, Susanne Schüler-Toprak, Barbara Schmalfeldt, Annette Hasenburg

**Affiliations:** 1 Department of Obstetrics and Gynecology, University Medical Center, Johannes Gutenberg University Mainz, Langenbeckstraße 1, 55131 Mainz, Germany; 2 Department of Gynecology and Obstetrics, University Hospital Ulm, Prittwitzstr. 43, 89075 Ulm, Germany; 3 Department of Gynecology and Obstetrics, University Hospital Leipzig, Liebigstraße 201, 04177 Leipzig, Germany; 4 Alb-Donau Klinikum, Spitalstraße 29, 89584 Ehingen, Germany; 5 University Medical Center Regensburg, Comprehensive Cancer Center (CCC) Würzburg, Erlangen, Regensburg, Augsburg Germany; 6 Department of Gynecology and Obstetrics, National Center for Tumor Diseases (NCT), Landshuter Str. 65, Regensburg, Germany; 7 Department of Gynecology and Gynecologic Oncology, University Medical Center Hamburg-Eppendorf, Hamburg, Germany; 8 Frauenärztin Bettingen, Frenkinger Platz 1, 54646 Bettingen, Germany

**Keywords:** Sustainability, Climate change, Gynecology, Environmental awareness, Resource conservation

## Abstract

**Objective:**

To assess the level of awareness, attitudes, and implementation of sustainability practices among gynecologists in Germany, and to identify barriers to sustainable behavior within the field.

**Background:**

Climate change increasingly affects gynecological and obstetric care. The world health organization (WHO) and the International federation of gynecology and obstetrics (FIGO) have emphasized the need for stronger climate action in healthcare. In response, the German society for gynecology and obstetrics (DGGG) established a working group on sustainability. However, empirical data on sustainability awareness among gynecologists worldwide are lacking. Understanding current attitudes is essential for developing targeted strategies to enhance sustainability in clinical and academic settings.

**Methods:**

A cross-sectional, nationwide online survey was conducted from February to June 2024 in Germany using a self-developed questionnaire. The survey assessed sustainability awareness, perceived relevance of climate change, and implementation of environmentally sustainable measures in both clinical and private practice contexts. Descriptive and comparative statistical analyses were performed.

**Results:**

Most respondents reported a high level of awareness regarding climate change, with female participants rating their individual contribution more strongly. Sustainable measures such as waste separation and the reduction of single-use packaging were more frequently implemented in private practices than in hospitals. A large proportion of participants reported using environmentally friendly transportation and avoiding short-haul flights. Virtual participation in conferences was common, and many respondents expressed willingness to pay higher fees for sustainable conference formats. The main barriers to implementation were time constraints, financial limitations, and a lack of sustainable alternatives.

**Conclusion:**

Sustainability awareness among gynecologists in Germany is increasing, particularly in personal and outpatient practice contexts. Greater dissemination of information and stronger professional networks are needed to promote sustainable practices and strengthen the contribution of gynecology and obstetrics to climate protection in healthcare.

## Introduction

Climate change and its effects are becoming increasingly apparent in all spheres of life, extending to gynecological and obstetric care [1]. The global climate crisis presents physicians with new medical, ethical, and structural challenges. Leading medical organizations, including the world health organization (WHO) [2], and the German medical association [3] and the international federation of gynecology and obstetrics (FIGO) [4], have therefore called for stronger engagement in climate protection and the achievement of climate neutrality within the healthcare sector. In response to these calls, the German society for gynecology and obstetrics (DGGG) established a working group on “sustainability” [5]. Similar initiatives have been implemented in recent years by other professional societies [6–10], aiming to raise awareness of sustainability in clinical practice and to promote concrete measures within professional organizations and working groups. However, the extent of sustainability awareness among healthcare professionals remains unclear. In the field of gynecology and obstetrics, existing data are limited to a single survey conducted in the United States [10]. That study found that many gynecologists and obstetricians are aware of the relevance of climate change and particularly recognize waste generation as a major harmful factor. Nevertheless, it remains unclear whether concrete preventive measures have already been implemented in professional or private contexts. To date, no comparable data are available from Germany or other countries. To develop and implement evidence-based strategies promoting sustainability in gynecological and obstetric care in Germany, it is essential to assess the current status quo and the motivation of healthcare professionals to contribute. Insights from such data are crucial for the establishment of effective, sustainable national strategies and initiatives.

## Methods

### Questionnaire

To assess the current level of awareness and existing measures to enhance sustainability among gynecologists working in Germany, a nationwide survey was conducted. As no validated instruments for measuring climate awareness among physicians were identified in the literature, a new questionnaire was developed specifically for this purpose.

Members of the working group on sustainability of the German society for gynecology and obstetrics (DGGG) [5] designed a 20-item questionnaire tailored to gynecologists practicing in Germany. In a pilot phase, the questionnaire was reviewed by the working group and a small test group for content, structural logic, and plausibility. The questionnaire included items on sociodemographic variables (age, gender, number of children, and federal state), professional characteristics (type of patient care, workplace setting), and awareness of climate change in both professional and personal contexts. Additional questions addressed the implementation of sustainability measures in clinical and private environments (hospitals and private practices). Finally, participants were asked about their willingness to incur additional costs to support climate-friendly conference formats. Responses were recorded using multiple-choice options and seven-point Likert scales. To disrupt habitual response patterns, the response formats were varied.

### Recruitment

The online questionnaire was available for slightly more than three months (February 19–June 5, 2024) via the SurveyMonkey^®^ platform. The survey link was distributed through the DGGG newsletter, the professional journal “Der Frauenarzt”, social media (Instagram^®^), academic presentations, and private networks. Eligible participants included all gynecologists currently practicing in Germany.

### Statistical analysis

Data underwent preliminary plausibility checks and cleaning prior to analysis. Participant characteristics were described using descriptive statistics, including medians, ranges, and absolute and relative frequencies. Correlations were calculated to assess relationships between variables, and subgroup differences were analyzed using the Chi-square test. A two-sided significance level of *p* < 0.05 was considered statistically significant. Internal consistency of Likert-scale blocks was assessed using Cronbach’s alpha.

## Results

### Cohort characteristics

Over a data collection period of 107 days, a total of 528 responses were analyzed. The proportion of female participants was 85%, male 14.2%, and 0.8% identified as diverse. The mean age of respondents was 44 years; female participants were significantly younger than their male colleagues. Approximately 30% of respondents were childless. Among those with children (39%), most reported having school-aged children. More than half of respondents (52.3%) worked in a hospital setting, with 44% of these employed at a university hospital. Among hospital-based participants, more than one-third (37.9%) held senior positions (consultant or head of department). In contrast, 44% of respondents worked in private practices or medical care centers (MVZ), of whom 64% were practice owners (see Table 1).

**Table 1:** Sociodemographic variables

Category	Subcategory	*n*
Gender	female	449 (85,0%)
	male	75 (14,2%)
	Diverse	4 (0,8%)
Age	Median (± SD)	44 Jahre (± 11,4)
	Females	42,6 Jahre
	Males	50,6 Jahre
Children	Childless	157 (29,7%)
	Newborn	11 (2,1%)
	Toddler	107 (20,3%)
	Schoolchild	206 (39,0%)
	In training	96 (18,2%)
	Employed	79 (15,0%)
Workplace	Clinic	276 (52,3%)
	Medical care center	26 (4,9%)
	Practice	201 (38,1%)
	Other	25 (4,7%)
Clinic	University hospital	134 (44,0%)
	Maximum care hospital	35 (11,4%)
	Municipal hospital	53 (17,3%)
	Private institution	37 (12,1%)
	Church institution	47 (15,4%)
	Not working at a hospital	222 (42,1%)
Job title	Chief physician	30 (5,7%)
	Senior physician	81 (15,3%)
	Specialist physician	69 (13,1%)
	Physician in further training	113 (21,4%)
	Practice owner	129 (24,4%)
	Employed in a practice	52 (9,9%)
	Student	6 (1,1%)
	Other	48 (9,1%)
Federal state	Baden-Württemberg	67 (12,3%)
	Bayern	63 (11,9%)
	Berlin	18 (3,4%)
	Brandenburg	7 (1,3%)
	Bremen	6 (1,1%)
	Hamburg	6 (1,1%)
	Hessen	62 (11,7%)
	Mecklenburg-Vorpommern	5 (1,0%)
	Niedersachsen	24 (4,6%)
	Nordrhein-Westfalen	104 (19,7%)
	Rheinland-Pfalz	45 (8,5%)
	Saarland	7 (1,3%)
	Sachsen	59 (11,1%)
	Sachsen-Anhalt	24 (4,6%)
	Schleswig–Holstein	26 (4,9%)
	Thüringen	5 (1,0%)

### Environmental awareness

A total of 73% of the participants fully agreed with the statement that society is currently experiencing an era of climate change (7/7 points on the Likert scale). The perceived influence of one’s own behavior on climate change—both privately and professionally—was rated as moderate on average (4.4/7 points). Sustainability was rated as more important in private life (5.6/7 points) than in professional contexts (4.8/7 points). Female participants rated their personal influence on climate change significantly higher than male participants (4.5 vs. 4.0 points; *p* < 0.001). Similarly, climate change was considered more relevant by women both in private life (5.7 vs. 5.2 points; *p* < 0.001) and in professional life (4.9 vs. 4.6 points; *p* = 0.012) (see Table 2). Regression analyses showed that other potential influencing factors—such as age, professional position, or parental status—had no significant effect on responses. The most important motivators for climate-conscious and sustainable behavior were concern for future generations (mean 6.2 ± 1.3), personal conviction (5.9 ± 1.3), and moral conscience (5.8 ± 1.4). Financial incentives (3.6 ± 1.8) or religious belief (2.6 ± 1.9) played a minor role.

**Table 2:** Awareness

	MW (± SD); Med	Influencing factor: gender
“We are currently living in an age of climate change.”	6.3 (± 1.5) LP	*p* < 0,001
	Female: 6.4 (± 1.4) LP	
	Male: 5.8 (± 1.8) LP	
“How strong do you think the influence of your personal behavior in your private or professional life is on climate change?”	4.4 (± 1.4); 5	*p* < 0,001
	Female: 4.5 (± 1.3) LP	
	Male: 4.0 (± 1.7) LP	
“How important is sustainability in your personal life?”	5.6 (± 1.3) LP	*p* < 0,001
	Female: 5.7 (± 1.3) LP	
	Male: 5.2 (± 1.6) LP	
“How important is sustainability in your professional life?”	4.8 (± 1.6)	*p* = 0,012
	Female: 4.9 (± 1.6) LP	
	Male: 4.6 (± 1.7) LP	

*SD* Standard deviation, *LP* Likert scale points

### Sustainable practices

**Fig. 1:**
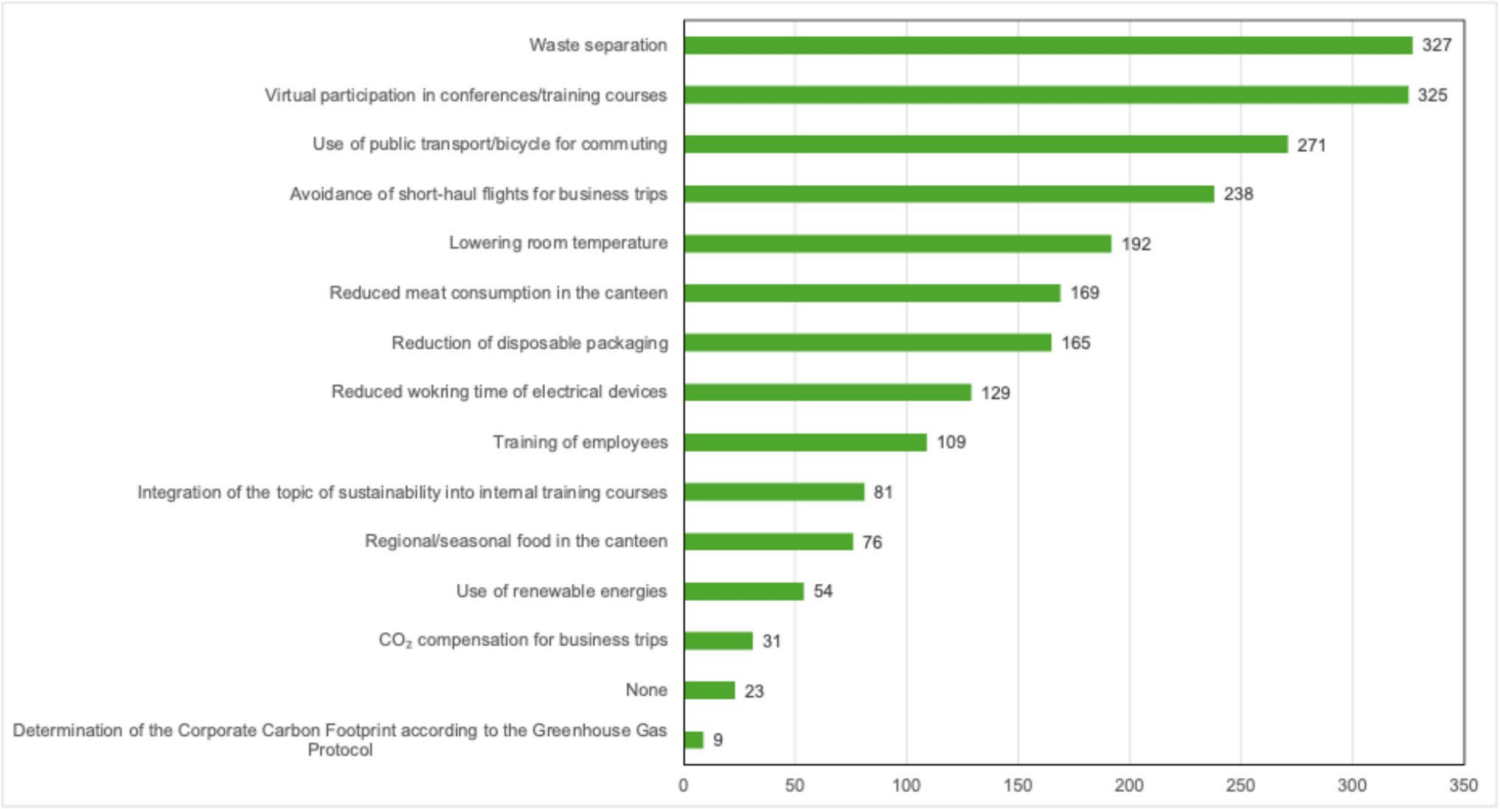
Which measures do you implement in your professional practice to live more sustainably?

Sustainable measures were implemented significantly more often in private practices than in hospital settings (57.2% vs. 49.0%; *p* < 0.001). Overall, waste separation was the most frequently reported sustainability measure (65%) (see Fig. 1).

Nevertheless, waste separation was reported significantly less often in the professional context than in private life (65% vs. 97.5%; *p* < 0.001). Physicians working in private practices reported higher rates of waste separation (73.0% vs. 54.6%; *p* < 0.001), reduction of single-use packaging (41.9% vs. 23.5%; *p* < 0.001), and lowering of indoor temperature (45.5% vs. 29.5%; *p* < 0.001) compared with their colleagues in hospital settings. Environmentally friendly mobility measures were frequently implemented overall: 53.9% of all participants used public transport or bicycles for commuting, and 47.3% avoided short-haul flights for work-related travel. However, compensation for CO₂ emissions from business travel was rarely utilized (6.2%). Measures to raise sustainability awareness were comparatively less common: staff training (21.7%), integration of sustainability topics into internal professional development (16.1%), and calculation of the corporate carbon footprint (CCF) according to the greenhouse gas protocol (1.8%) were reported only sporadically (see Fig. 1). Recycling and the use of renewable energy were identified by respondents as particularly relevant strategies for increasing sustainability (see Fig. 4).

**Fig. 2:**
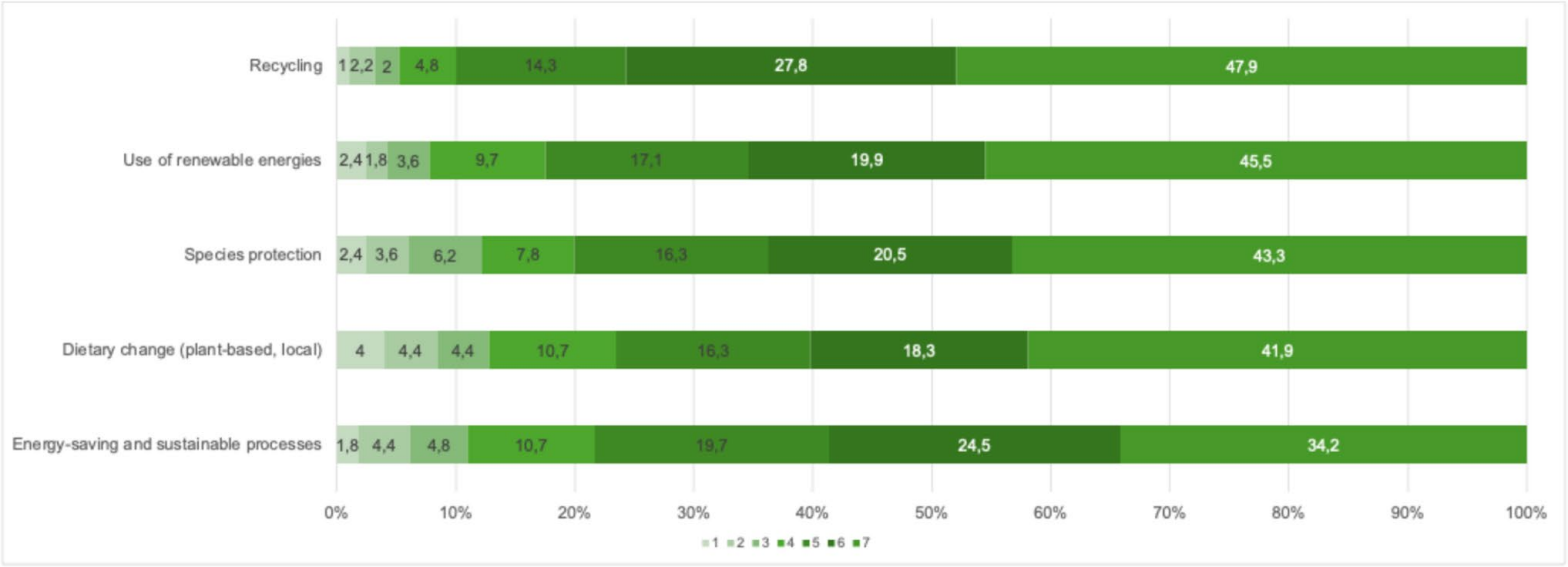
Factors limiting the implementation of sustainable practices in healthcare, as perceived by respondents (1 = not at all; 7 = strongly)

**Fig. 3:**
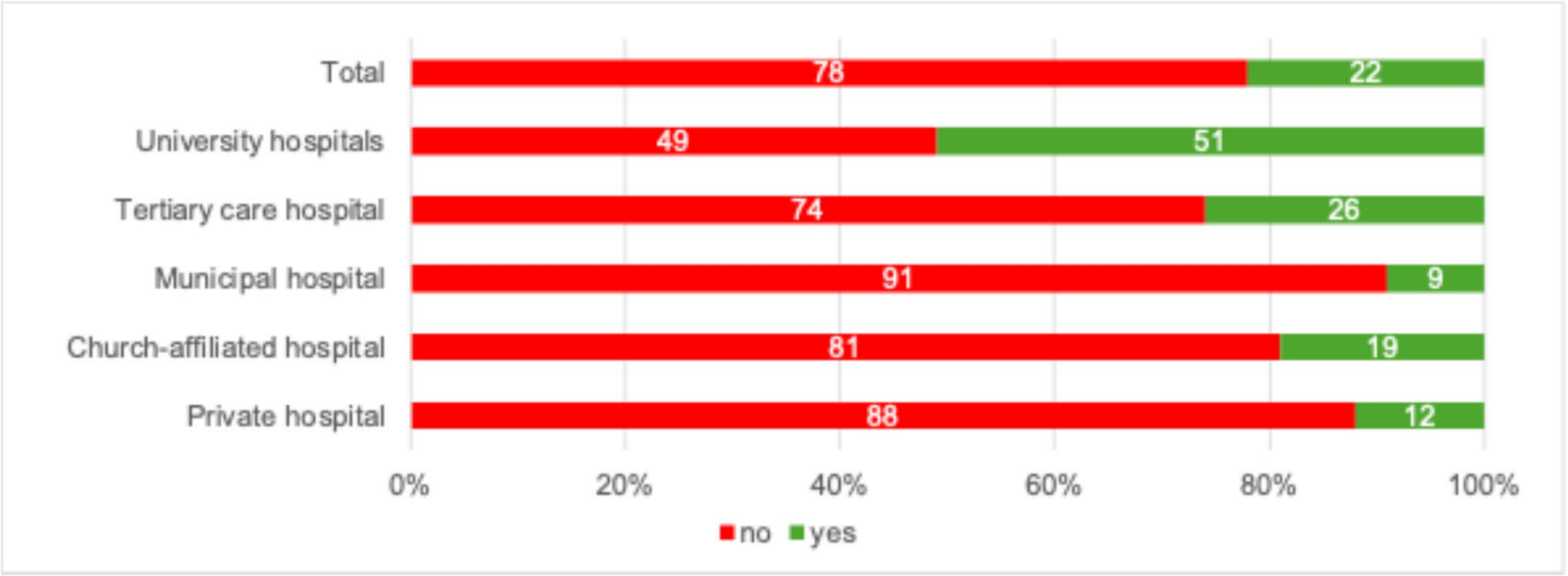
Presence of a sustainability working group, Green Team, or climate manager in respondents’ hospital or practice

A so-called “Green Team” or a comparable sustainability working group was established in 21.9% of institutions, most frequently at university hospitals (see Fig. 3). Institutions with such a committee implemented sustainability measures significantly more often than those without (88% vs. 45.8%; *p* < 0.001).

### Barriers

The main barriers to implementing a sustainable healthcare system were identified as time and cost constraints (5.5/7 points), lack of sustainable alternatives (5.3/7 points), and concern over potential financial losses (5.3/7 points). In addition, insufficient digitalization (5.2/7 points) and lack of knowledge (5.3/7 points) were perceived as limiting factors (see Fig. 2).

**Fig. 4:**
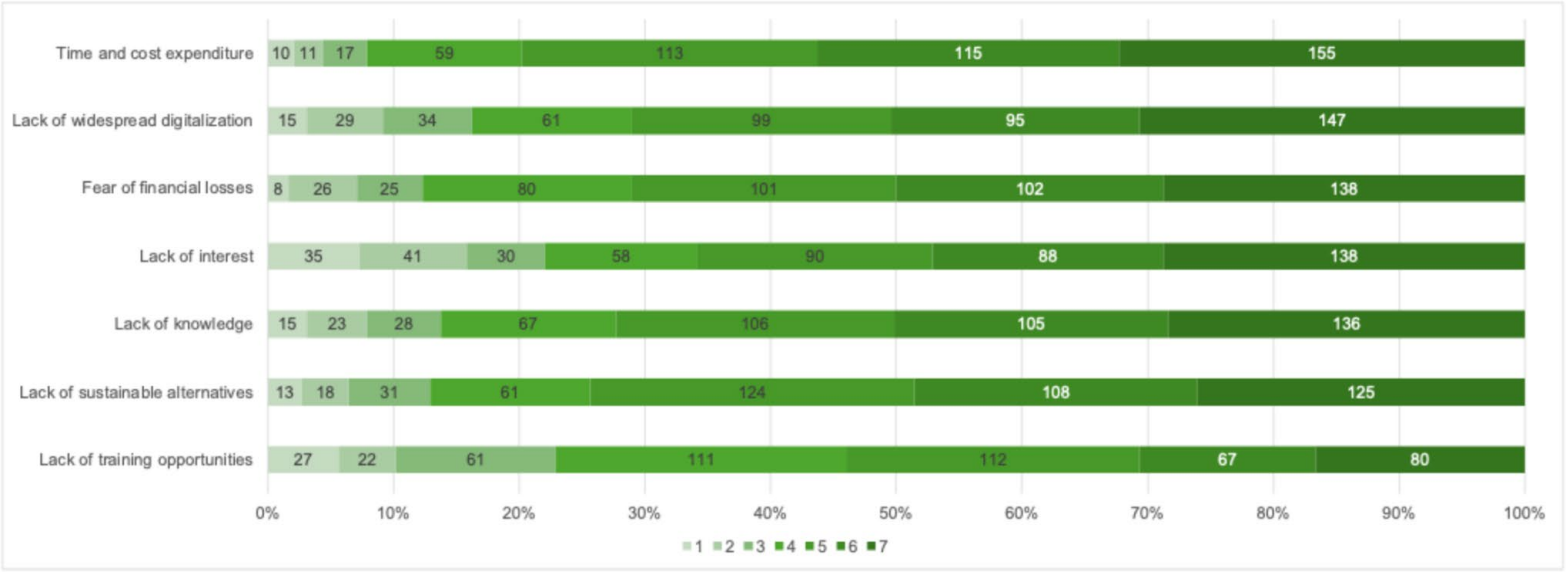
Motivating factors for engagement with sustainability (1 = no motivation; 7 = strong motivation)

### Knowledge and sources of information

**Fig. 5:**
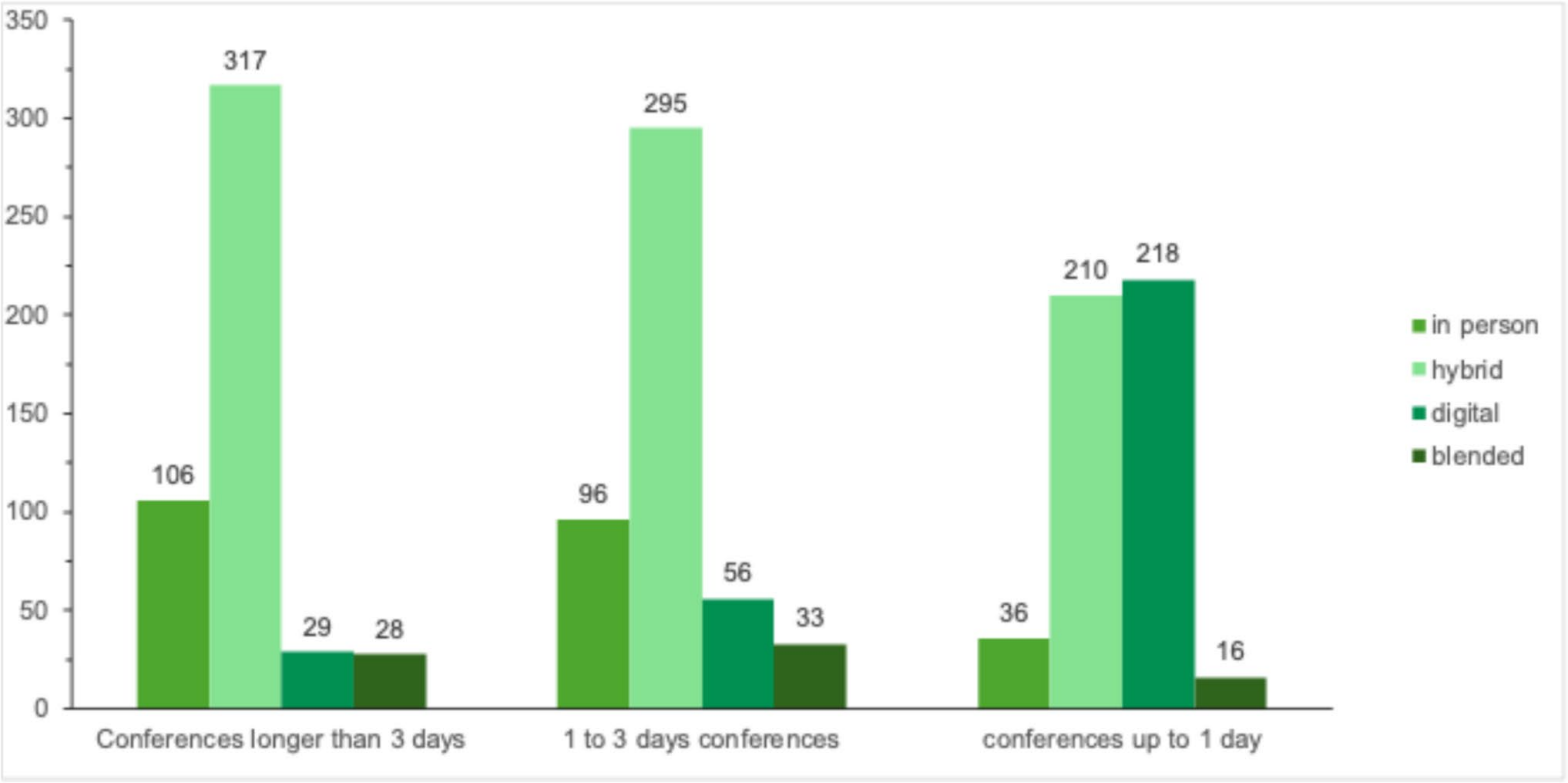
Respondents’ preferences for future event and conference formats

Participants rated their own knowledge of the effects of climate change on their specialty at an average of 3.6 ± 1.5 points, corresponding to a lower-intermediate level. Only 4% of the respondents considered their knowledge in this area to be “very good.” Knowledge about sustainability was primarily obtained from digital and media sources: 82.7% cited the internet, 69.4% print media, and 52.0% family or friends as information sources. Specialty-specific conferences and continuing education contributed to knowledge acquisition for 41.6% of respondents. In contrast, sustainability content in medical education or training played a minor role; only 6.0% reported having received relevant content during their formal education (see Table 3) (Fig. 5).

**Table 3:** Knowledge acquisition

Where do you get your knowledge about sustainability?	% (*n*)
Conferences/training courses	41,6% (207)
Newspapers/magazines	69,5% (346)
Internet	82,7% (412)
Social media	38,8% (193)
Television	43,4% (216)
Education	6% (30)
Family/friends	52% (259)
Other	3,8% (19)

### Conferences

A large proportion of respondents (64.6%) reported attending conferences or continuing education events virtually to reduce emissions from travel. Attitudes toward willingness to pay higher fees for more sustainable event formats were heterogeneous: 46.3% were willing to pay up to 50% higher fees, while 15% would pay double. At the same time, 36.7% of respondents completely rejected any cost increase in the name of sustainability. Regarding preferred event formats, there was a clear tendency toward hybrid conferences. Hybrid formats were favored for multi-day events (over three days) by 66% of respondents and for shorter events (one to three days) by 61.5%. In-person formats were considerably less preferred (over three days: 22.1%; one to three days: 20%). Notably, when asked about future preferences, respondents indicated a shift away from purely physical conferences (7.5%) toward hybrid (43.8%) or fully digital events.

## Discussion

Our study represents the first data collection on sustainability awareness among gynecologists in Germany. The surveyed cohort reflects the current structure of the specialty, which is characterized by a predominance of female physicians, and includes participants from both clinical and outpatient settings across all age groups, life situations, and professional positions. The data indicate a high level of environmental awareness among respondents, with more than three-quarters acknowledging that they are currently living in an era of climate change. Sustainability is particularly important in private life but is also actively considered in professional practice.

Previous surveys support these findings. A survey by the Hartmann Association, including 283 physicians from all specialties in Germany, reported that approximately 75% of respondents considered the healthcare sector relevant to climate change, and 85% considered themselves climate-conscious [11]. Similarly, a survey by the association of specialist physicians in Germany found that 78% of respondents assigned high importance to sustainability [12]. Comparable results were reported in a U.S. study, where 73.1% of physicians indicated that sustainability was relevant [13]. These findings suggest that awareness of sustainability is generally present, though the implementation of concrete measures appears heterogeneous in the literature.

In our cohort, more than 20% of respondents reported the presence of a Green Team. Measures such as establishing Green Teams or appointing climate managers can help centralize actions to enhance sustainability within institutions and have been shown to be effective in other studies [14]. Among concrete climate-friendly measures, respondents most commonly reported self-directed actions, such as using public transport or cycling, as well as waste separation and recycling. These measures are both autonomous and easy to implement, which facilitates their adoption—a finding also reported by other authors [15]. Shared housing or vegetarian diets have also been described as frequent sustainability measures among physicians [15]. In contrast, centrally coordinated measures, such as the use of renewable energy or calculation of the corporate carbon footprint (CCF), were rarely implemented according to our survey. Data on the frequency of CCF calculation in Germany or other countries within healthcare settings are not currently available [16, 17]. Intrinsic motivation plays an important role in promoting sustainability [18, 19], which was confirmed in our cohort. Concern for future generations, personal conviction, and moral conscience were relevant motivators, while religious affiliation played no significant role. International data also indicate that conviction and conscience are important factors supporting sustainability initiatives [18].

Respondents identified time and cost constraints as major barriers, as sustainable measures are rarely part of standard work tasks and therefore require additional resources. Other limiting factors included insufficient digitalization, lack of knowledge, and the absence of sustainable alternatives. External institutional barriers were particularly relevant, as noted in our cohort and in other studies [15, 16, 20]. Regarding knowledge and awareness of sustainable alternatives, further research and education are needed to ensure that truly sustainable practices are implemented. Existing reviews provide guidance for clinical daily practice [21–24], but only partially reflect the full scope of routine clinical activities.

Conferences represent an essential component of continuous medical education beyond daily clinical work. In addition to scientific exchange, they provide opportunities to expand professional networks, engage in face-to-face interactions, discuss latest developments with industry representatives, and collaborate within specialty and working groups. The importance of conference participation was reflected in respondents’ willingness to accept additional costs to attend climate-friendly events. The COVID-19 pandemic in 2020 led to the establishment of virtual and hybrid formats for continuing medical education. This development underscores the need to evaluate which conferences require physical attendance and for which formats digital participation is an adequate alternative. In our survey, 89.2% of participants preferred digital or hybrid attendance for one-day events, reducing travel costs and time, and yielding additional co-benefits [19]. For multi-day conferences, such as the biennial DGGG Congress, the pattern differs: 88.1% of respondents considered in-person or hybrid attendance highly important. According to Tao et al., [25] hybrid conferences can provide substantial sustainability benefits, a strategy already discussed and partially implemented for selected events in Germany and the U.S. [19, 26].

The strengths of our study lie primarily in its comprehensive scope, which, to our knowledge, represents the largest survey on sustainability in healthcare in Germany to date. We captured aspects of both private and professional life, identified relevant motivators and barriers, and evaluated participants’ readiness to engage in climate-conscious behaviors at conferences. Despite the relatively large sample size (*n* = 500), corresponding to approximately 2.5% of the target population (approximately 19.000–20.000 gynecologists in Germany), the semi-representative nature of the cohort warrants cautious interpretation of statistically significant findings, as these may not necessarily indicate effects of substantial magnitude or broad applicability. A potential selection bias should also be considered, as physicians with a strong interest in sustainability may have been more likely to participate, while those less engaged may be underrepresented. Consequently, the findings may be only partially representative, as respondents with a strong interest in sustainability are more likely to have already implemented corresponding changes and practices in both their professional and personal lives.

## Summary

There is an increasing awareness of sustainability among gynecologists in Germany, which is particularly evident in measurements taken in private life (5.6/7 points) compared with actions taken in professional practice (4.8/7 points). Regarding concrete measures, self-directed initiatives can often be implemented more quickly and are therefore more commonly adopted in private practices than in hospital settings (57.2% vs. 49.0%; *p* < 0.001). In larger institutions, such as hospitals, organizational barriers frequently hinder the rapid implementation of more sustainable processes. The data underscore a substantial demand for guidance on sustainable practices, given that participants’ average self-assessed knowledge was only at a lower-intermediate level (3.6/7 points). Through networking and collective efforts, gynecologists in Germany can more effectively contribute to promoting sustainability, with a focus on the needs of future generations.

## Data Availability

No datasets were generated or analyzed during the current study.
